# Painful Schwannoma Arising From the Great Auricular Nerve

**DOI:** 10.7759/cureus.108055

**Published:** 2026-04-30

**Authors:** Mikio Shimazaki, Masami Suzuki, Naohiro Yoshida

**Affiliations:** 1 Department of Otolaryngology-Head and Neck Surgery, Jichi Medical University Saitama Medical Center, Saitama, JPN

**Keywords:** cervical schwannomas, external jugular vein, great auricular nerve, great auricular nerve schwannoma, infra-auricular region, painful schwannoma, postauricular region, schwannoma

## Abstract

We report a case of a painful cervical schwannoma arising from the great auricular nerve (GAN) in an 84-year-old man. He presented with a long-standing left cervical mass and progressively worsening severe pain in the infra-auricular and postauricular regions. Physical examination and magnetic resonance imaging revealed a mass along the sternocleidomastoid muscle coursing parallel to the external jugular vein. Based on the characteristic pain distribution and anatomical findings, a schwannoma arising from the GAN was suspected preoperatively. Complete tumor excision with transection of the GAN was performed for pain relief, and histopathological examination confirmed the diagnosis of schwannoma. Postoperatively, the pain resolved completely, with no recurrence during 18 months of follow-up. This case underscores the diagnostic value of characteristic pain distribution and anatomical features in the preoperative identification of a schwannoma arising from the GAN.

## Introduction

A schwannoma is a benign peripheral nerve sheath tumor that commonly occurs in the head and neck region [1]. Cervical schwannomas usually arise from mixed or autonomic nerves, including the vagus nerve, sympathetic chain, and brachial plexus, and typically present as slow-growing, painless cervical masses [1-3]. Because of their indolent course, these tumors often produce few or no symptoms, making preoperative identification of the nerve of origin challenging. Schwannomas arising from the great auricular nerve (GAN), a purely sensory branch of the cervical plexus, are rare, with only a few cases reported [4-8]. The GAN is a superficial sensory nerve that courses parallel to the external jugular vein (EJV) and supplies the auricular, infra-auricular, and postauricular regions [9,10]. Clinically, these lesions typically present as slowly enlarging masses in the lateral neck or periauricular region, reflecting the cutaneous distribution of the GAN. Most reported GAN schwannoma cases have presented as painless cervical masses [5-8], although pain within the sensory distribution of the GAN has occasionally been described [4]. We report a rare case of a painful cervical schwannoma arising from the GAN in an elderly man. This case highlights the clinical pain distribution and anatomical relationship to the EJV as useful clues for the preoperative diagnosis of GAN schwannoma, thereby improving recognition of the nerve of origin and guiding surgical decision-making for this uncommon lesion.

## Case presentation

**Figure 1:**
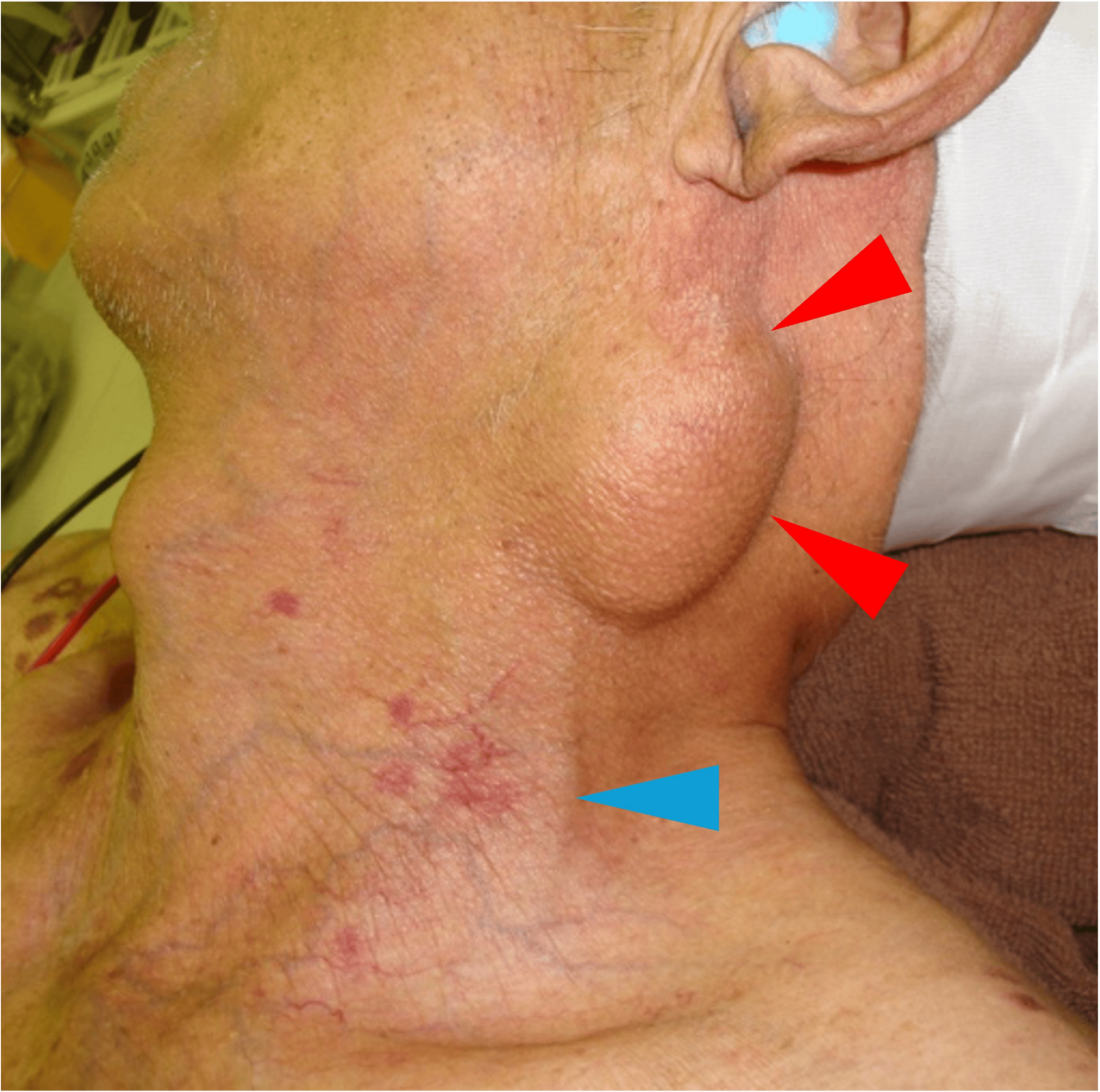
Left cervical mass adjacent to the external jugular vein Red arrow: mass; blue arrow: external jugular vein

An 84-year-old man had first noticed a left cervical mass approximately 50 years earlier, and it had gradually enlarged over time. Nine years prior to presentation, he developed pain below and posterior to his left ear, and the pain progressively worsened, prompting referral to our department. His medical history included chronic renal failure requiring maintenance hemodialysis, prior aortic valve replacement, thoracic aortic aneurysm surgery, and ongoing anticoagulant therapy. Physical examination revealed a mobile, 40-mm mass in the left cervical region adjacent to the EJV (Figure 1). No sensory deficits, skin changes, or other neurologic abnormalities were observed in the cutaneous distribution of the GAN.

**Figure 2:**
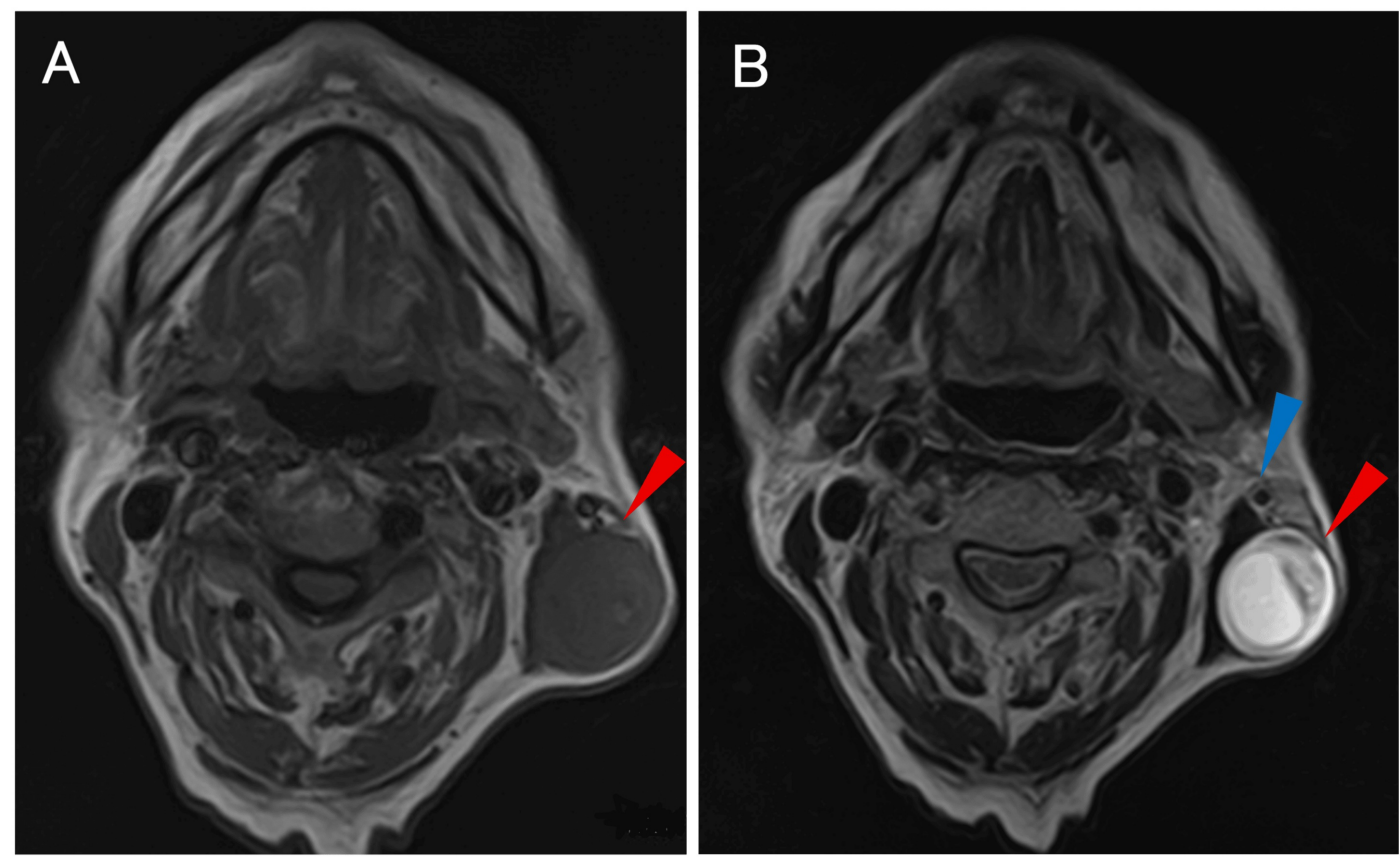
MRI findings (A) Axial T1-weighted image showing a mass with an isointense signal relative to muscle (red arrow). (B) Axial T2-weighted image showing a mass with high signal intensity and mild internal heterogeneity (red arrow), adjacent to the external jugular vein (blue arrow). MRI: magnetic resonance imaging

Resting (baseline) pain was rated 8/10 on the numerical rating scale (NRS) prior to palpation, and palpation elicited severe pain extending from the infra-auricular to the postauricular region. Plain cervical magnetic resonance imaging (MRI) showed a 40-mm mass along the left sternocleidomastoid muscle (SCM), posterior to the EJV. Contrast-enhanced MRI was not performed due to the patient’s chronic renal failure. The lesion demonstrated low to isointense signal intensity relative to muscle on T1-weighted images and high signal intensity with mild internal heterogeneity on T2-weighted images, findings suggestive of a neurogenic tumor (Figure 2).

Ultrasonography was not performed due to severe pain. The characteristic pain distribution, superficial location over the SCM, MRI findings, and proximity to the EJV raised suspicion of a GAN schwannoma.

For pain relief, complete tumor resection with nerve transection was chosen over subcapsular excision, as subcapsular dissection along the tumor capsule may encounter feeding vessels and make hemostasis difficult; therefore, complete resection was considered to shorten operative time and reduce bleeding risk in the setting of anticoagulation and hemodialysis.

**Figure 3:**
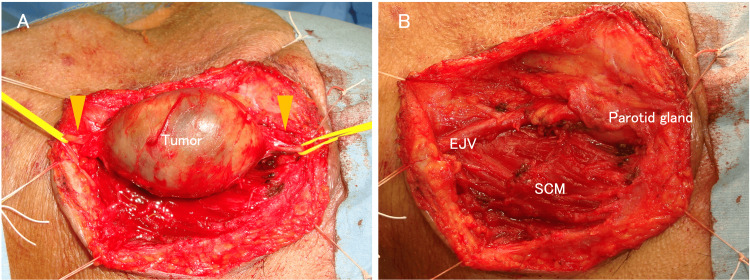
Operative findings (A) Before tumor resection; yellow arrow: the great auricular nerve. (B) After tumor resection. SCM: sternocleidomastoid muscle, EJV: external jugular vein

A skin incision was made over the tumor, and flaps were elevated beneath the platysma. The tumor was situated parallel to the EJV and was found to be in continuity with the GAN both proximally and distally (Figure 3A). The proximal and distal segments of the GAN were transected, and the tumor was subsequently excised (Figure 3B). 

**Figure 4:**
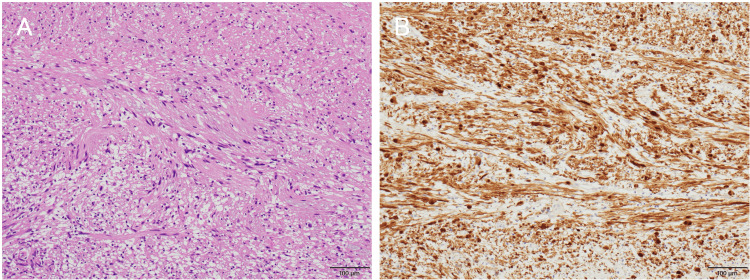
Histopathological findings (A) Hematoxylin and eosin staining showing proliferation of spindle-shaped cells with minimal nuclear atypia (original magnification ×100). (B) Immunohistochemical staining for S-100 protein showing diffuse strong positivity in tumor cells (original magnification ×100).

The tumor measured 46 mm in its greatest dimension. Histopathological examination revealed spindle-shaped cells with minimal atypia (Figure 4A). Immunohistochemical staining showed diffuse positivity for S-100 protein, consistent with schwannoma (Figure 4B).

On postoperative day (POD) 1, the NRS score improved to 0/10 after tumor excision, and no analgesic medications were required postoperatively. Earlobe numbness was noted, consistent with GAN transection. Intraoperative bleeding was minimal; however, the wound required compression for seven days postoperatively because of persistent subcutaneous hemorrhage. Anticoagulation therapy was continued due to underlying cardiovascular disease. No additional intervention, such as imaging or drainage, was required. Discharge was slightly delayed until adequate hemostasis was achieved. The patient was discharged on POD 10. At 18 months postoperatively, the sensory deficit had slightly improved but remained partially persistent, and pain relief was sustained without recurrence.

## Discussion

A cervical schwannoma is a benign tumor that originates most commonly from mixed or autonomic nerves, including the vagus nerve, sympathetic chain, and brachial plexus. Because it grows slowly, it often produces few or no subjective symptoms [1-3]. A schwannoma arising from the GAN, a purely sensory nerve, is rare. We conducted a literature search of English-language articles in PubMed and Google Scholar, and Japanese-language articles using Ichushi-Web, for cases of cervical schwannomas arising from the GAN, using the terms “great auricular nerve schwannoma” and “GAN schwannoma,” with the last search conducted on March 26, 2026. Including the present case, only six cases have been reported (Table 1) [4-8].

**Table 1:** Characteristics of reported cases of great auricular nerve schwannomas * The lesion was initially diagnosed as a sebaceous cyst at another institution. During surgery under local anesthesia, infra-auricular pain occurred, raising suspicion of a schwannoma of the great auricular nerve, and the procedure was discontinued. Subsequently, nerve-sparing excision was performed under general anesthesia. ** Postoperative pain was not reported in cases without preoperative pain.

Author	Year	Age (years)	Sex	Preoperative pain	Size (mm)	Preoperative diagnosis	Surgical procedure	Postoperative pain
Fukazawa et al. [4]	2008	66	M	Yes	15	Schwannoma (great auricular nerve origin)	Tumor and nerve resection	No
Xu et al. [5]	2019	29	F	No	15	Schwannoma (nerve of origin unknown)	Nerve-sparing excision	Not reported**
Oh et al. [6]	2021	76	F	No	20	Not reported	Tumor and nerve resection	Not reported**
Mizokami et al. [7]	2024	40	M	No	7	Sebaceous cyst*	Nerve-sparing excision	Not reported**
Fotiadou et al. [8]	2025	26	F	No	21	Sebaceous cyst	Tumor and nerve resection	Not reported**
Present case	2026	84	M	Yes	46	Schwannoma (great auricular nerve origin)	Tumor and nerve resection	No

Among the reported cases, the mean age was 54 years, the male-to-female ratio was 1:1, and two cases presented with pain; however, these findings should be interpreted with caution, given the small number of cases.

This case highlights three important clinical lessons.

First, a GAN schwannoma may present with severe pain, which can improve after surgical excision. Pain associated with schwannomas is thought to result from compression of the nerve fibers by the tumor; therefore, pain often resolves after tumor excision [11,12]. In the two reported cases with pain, tumor resection resulted in symptomatic improvement. Surgical treatment for cervical schwannomas includes complete resection with division of the nerve fibers and tumor excision with preservation of the nerve fibers [2,13]. From a nerve-sparing perspective, tumor resection with nerve preservation is generally recommended. However, complete resection may be considered in cases where a definitive diagnosis can be made preoperatively because GAN resection is unlikely to significantly impair the quality of life of the patient [14,15]. Potential sensory symptoms, such as numbness or hypoesthesia in the auricular and infra-auricular regions, should be discussed with the patient preoperatively. In this case, given the patient’s advanced age and poor general condition, including ongoing anticoagulant therapy and end-stage renal disease requiring hemodialysis, complete resection was chosen to shorten the operative time and reduce the risk of intraoperative bleeding.

Second, a schwannoma arising from the GAN can be diagnosed preoperatively when a mass over the SCM is accompanied by pain in the sensory distribution of the GAN. In general, identification of the nerve of origin of cervical schwannomas is difficult based on imaging findings or histological examinations [1,16,17]. In contrast, in the reported cases (Table 1), preoperative diagnosis of a schwannoma arising from the GAN was possible in both cases presenting with pain. The GAN is the major sensory nerve supplying the angle of the mandible, the earlobe, and the infra-auricular and postauricular regions, and pain in these areas is considered a characteristic feature of GAN-related pain [9,10]. Therefore, when a mass is identified over the SCM in association with pain in these regions, a GAN schwannoma should be strongly suspected preoperatively; however, the diagnosis remains presumptive until surgical and histopathologic confirmation.

**Figure 5:**
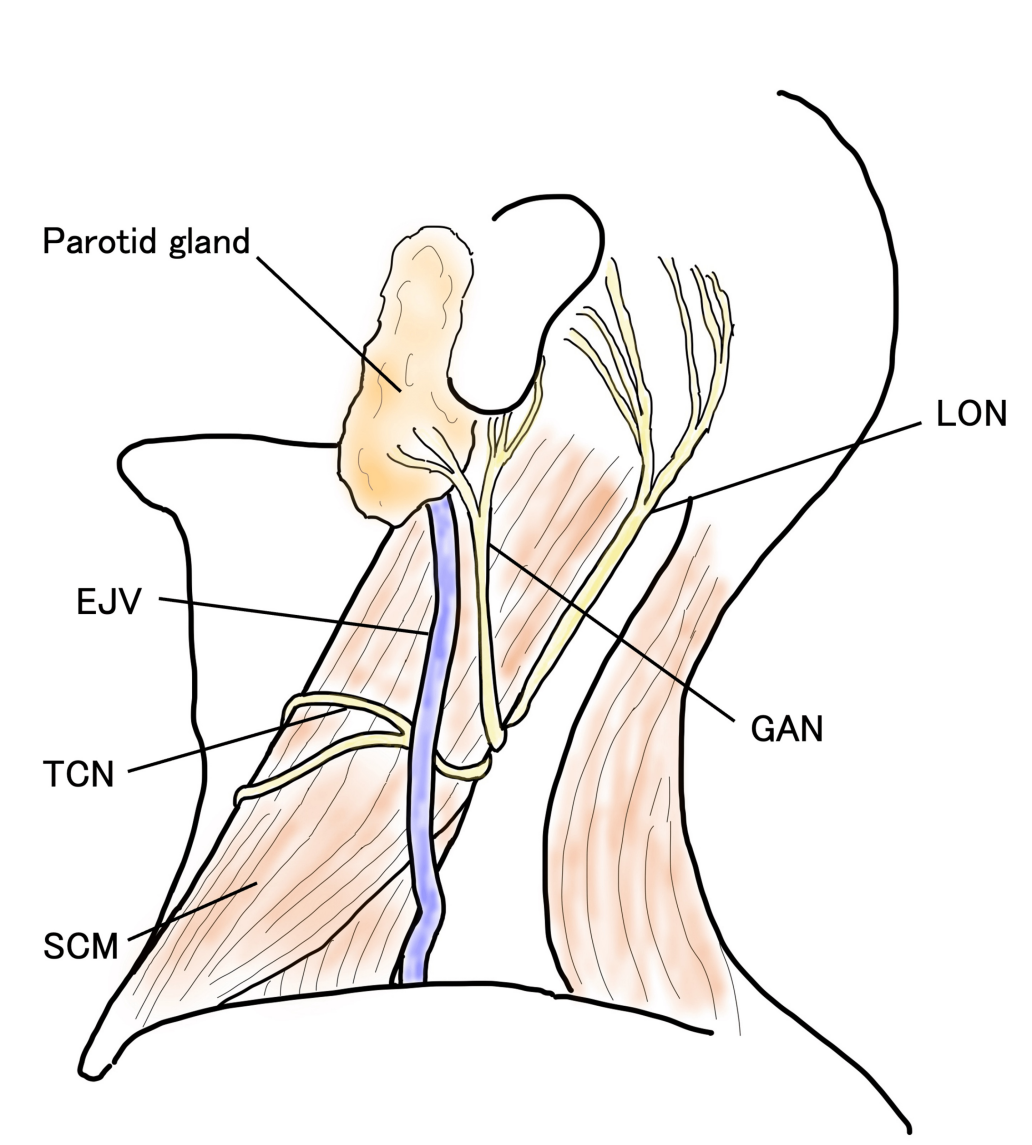
Schematic illustration of the left lateral neck showing the cutaneous cervical nerves and the EJV over the SCM The GAN accompanies the EJV over the SCM. SCM: sternocleidomastoid muscle, EJV: external jugular vein, GAN: great auricular nerve, LON: lesser occipital nerve, TCN: transverse cervical nerve This figure is an original illustration created by the authors using GoodNotes (Goodnotes Limited, London, United Kingdom) and Procreate (Savage Interactive Pty Ltd., Tasmania, Australia).

Third, in painless masses over the SCM, focusing on the anatomical feature that the GAN courses in parallel with the EJV may allow a preoperative diagnosis. In GAN-derived schwannomas, the occurrence of pain varies depending on the tumor's progression pattern. As shown in Table 1, schwannomas arising from the GAN often present as a cervical mass even in the absence of pain, and surgical excision may be selected depending on the patient’s preference. To anticipate the potential risk of postoperative neurological deficits, it is important to establish the most accurate preoperative diagnosis. The nerves that may give rise to schwannomas occurring over the SCM include the GAN, lesser occipital nerve (LON), and transverse cervical nerve (TCN) (Figure 5) [10].

The GAN branches from the anterior rami of the C2-C3 cervical plexus, emerges subcutaneously at the midpoint of the posterior border of the SCM (Erb’s point), and ascends parallel to the EJV [9,10]. The LON appears over the SCM at the upper two-thirds of its posterior border and courses superiorly along the posterior border of the muscle [10]. The TCN emerges over the SCM at the level of Erb’s point and courses horizontally in an anterior direction [10]. Among these three nerves, a characteristic feature of the GAN is its upward course parallel to the EJV. In Cases 1-5, imaging studies demonstrated that the tumors were located parallel to the EJV. In painless cases, localization of the tumor parallel to the EJV may serve as a useful diagnostic clue for suspecting a GAN schwannoma. Although preoperative diagnosis was relatively straightforward in the present case because of the presence of pain, the parallel localization of the tumor and the EJV on physical examination (Figure 1) and imaging findings (Figure 2A) would also have allowed a preoperative diagnosis.

Schwannomas typically appear on MRI as well-circumscribed, encapsulated masses. On T1-weighted images, they demonstrate iso- to slightly low signal intensity relative to muscle, whereas on T2-weighted images, they show high signal intensity and often exhibit internal heterogeneity [18]. On ultrasonography, schwannomas typically appear as well-defined fusiform or ovoid hypoechoic masses. The internal echotexture is usually homogeneous to mildly heterogeneous and may become more heterogeneous with tumor enlargement due to cystic or degenerative changes. Continuity with the parent nerve at both ends of the mass (the entering and exiting nerve sign) may be observed and is helpful for diagnosis [19,20]. Although it is not always possible to clearly identify the nerve of origin on imaging, these findings provide important clues suggestive of a neurogenic tumor. In the present case, MRI demonstrated a well-defined mass showing low to iso-intensity on T1-weighted images and high signal intensity on T2-weighted images, supporting the diagnosis of schwannoma and contributing to the preoperative diagnosis.

**Figure 6:**
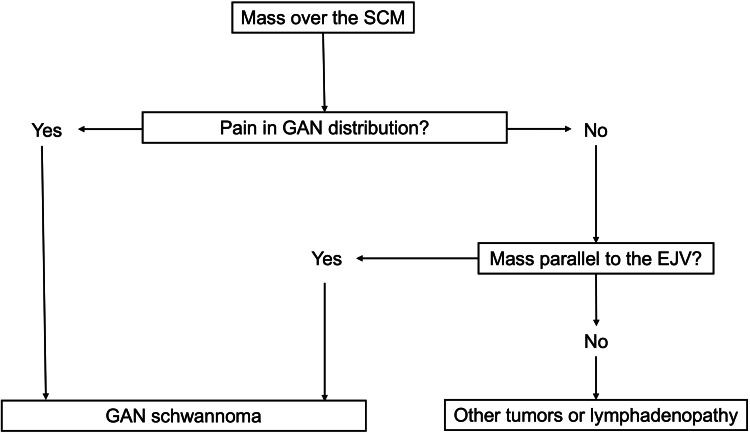
Flowchart of the preoperative diagnostic approach for a mass lesion over the SCM SCM: sternocleidomastoid muscle, GAN: great auricular nerve, EJV: external jugular vein This figure is an original illustration created by the authors using Microsoft PowerPoint (Microsoft Corporation, Redmond, WA, US).

The flow of preoperative diagnosis for a mass lesion over the SCM is illustrated in Figure 6. 

The presence of a painful mass in the angle of the mandible, earlobe, infra-auricular, or postauricular regions supports a preoperative diagnosis of GAN schwannoma. In the absence of pain, identification of a mass running parallel to the EJV supports the diagnosis of GAN schwannoma. Conversely, when a mass does not run parallel to the EJV, other diagnoses should be considered. Although simplified, this algorithm may serve as a useful clinical guide when considered in the context of a broad differential diagnosis, including lymphadenopathy, cystic lesions, salivary lesions, other peripheral nerve tumors, and Morel-Lavallée lesions.

## Conclusions

Schwannomas arising from the GAN may present with severe pain, and symptoms may improve after tumor excision; however, this observation is based on a limited number of cases and should be interpreted with caution. When pain involving the angle of the mandible, the earlobe, and the infra-auricular and postauricular regions is accompanied by a mass over the SCM, a preoperative diagnosis of GAN schwannoma may be possible. In painless masses over the SCM, focusing on the anatomical feature that the GAN courses in parallel with the EJV may allow a preoperative diagnosis.
